# A new perspective on word order preferences: the availability of a lexicon triggers the use of SVO word order

**DOI:** 10.3389/fpsyg.2015.01183

**Published:** 2015-08-13

**Authors:** Hanna Marno, Alan Langus, Mahmoud Omidbeigi, Sina Asaadi, Shima Seyed-Allaei, Marina Nespor

**Affiliations:** ^1^Neuroscience Sector, International School for Advanced Studies–SISSATrieste, Italy; ^2^School of Medicine, Shahid Beheshti University of Medical SciencesTehran, Iran; ^3^School of Cognitive Sciences, Institute for Research in Fundamental Sciences (IPM)Tehran, Iran

**Keywords:** language, linguistics, gestures, cognitive resources, word order, grammar, lexicon

## Abstract

Word orders are not distributed equally: SOV and SVO are the most prevalent among the world's languages. While there is a consensus that SOV might be the “default” order in human languages, the factors that trigger the preference for SVO are still a matter of debate. Here we provide a new perspective on word order preferences that emphasizes the role of a lexicon. We propose that while there is a tendency to favor SOV in the case of improvised communication, the exposure to a shared lexicon makes it possible to liberate sufficient cognitive resources to use syntax. Consequently SVO, the more efficient word order to express syntactic relations, emerges. To test this hypothesis, we taught Italian (SVO) and Persian (SOV) speakers a set of gestures and later asked them to describe simple events. Confirming our prediction, results showed that in both groups a consistent use of SVO emerged after acquiring a stable gesture repertoire.

## Introduction

Even though there are six logically possible ways of arranging words into sentences according to their basic grammatical functions of Subject, Object, and Verb (SVO, SOV, VSO, VOS, OVS, OSV), the SOV and SVO orders account for 86% of word order variation among the world's languages (Dryer, 2005). The preferences for these two specific word orders suggest that the triggers for some word order regularities might be genetically endowed in our cognitive repertoire. However, it is unknown why specific word order regularities emerge and precisely how they are triggered. The fact that 86% of natural languages have Subject first indicates that this is a common preference of language. The Subject in first position is also present in the expressions of homesigners, deaf children of hearing parents who have had no contact with any conventional language and in order to communicate invent their own gestural communication (Coppola and Newport, 2005). However, regarding the position of Verb and Object there is a larger diversity. While in the literature some people have proposed that SOV might be the order that emerged first in early human languages (Givón, 1979; Newmeyer, 2000; Gell-Mann and Ruhlen, 2011), and that there is evidence in many European languages for a unidirectional change from SOV to SVO (for an overview see Newmeyer, 2000), the source of the relative frequency of SVO in the current languages of the world is still a subject of a debate.

To explain the factors triggering preference for SOV and SVO, respectively, most theories emphasize semantic considerations such as saliency (Goldin-Meadow et al., 2008); confusability (Meir et al., 2010; Gibson et al., 2013); potential role-conflict between the communicator of a certain action-event and the agent of an action (Hall et al., 2013); or the abstractness of the event (Schouwstra et al., 2011; Schouwstra, 2012). A common feature of these studies is that they use elicited pantomime, and that they vary the semantic features of the scenes that the participants are invited to gesture. However, while in the case of semantically simpler scenes the general finding is an explicit and well-defined preference for using the SOV word order, when more abstract or cognitively more demanding scenarios are presented, the distribution of word orders during gesturing becomes rather incoherent.

It has been argued that reversible situations, where also the patient of the action could take the role of the agent (e.g., the boy kicks the girl vs. the girl kicks the boy), may evoke a cognitive-functional pressure on the communicator (Hall et al., 2014). When a communicator is trying to describe a certain event by using gestures, s/he will automatically take the role of the agent of the action. Therefore, when s/he has to describe the patient of the event, which is another potential agent, this might lead to a *role-conflict* between the two elements of the event. The authors propose that a solution to this problem would be to switch from the basic word order (SOV) to the more efficient SVO, in which the Subject is separated from the Object by the Verb, or to SOSV by repeating the Subject before gesturing the Verb. The role-conflict hypothesis has been investigated by using elicited pantomime where both English (SVO) and Turkish (SOV) speakers used SOV during the description of non-reversible events (Hall et al., 2013, 2014). However, in the case of reversible events, while English speakers switched to SVO, Turkish speakers started to gradually increase their use of SVO only when they were also asked to be consistent in their gestures. Moreover, teaching these consistent gestures to a third person resulted in a significant increase of SVO, compared to their baseline condition. Importantly thus, reversibility alone appears to be insufficient to explain these results: also the transmission of a consistent gesture lexicon was needed to change word order preferences from SOV to SVO for speakers of an SOV language.

Alternatively, it has been argued that a preference for SVO could arise as a consequence of communicative-memory pressures in the case of reversible events (Gibson et al., 2013). According to this theory, both language production and language comprehension operate via a *noisy channel*. When the speaker attempts to convey a message across a channel, the message can be corrupted and it becomes difficult for the listener to understand. In the case of reversible events, where such corruption can emerge from the ambiguity of the agent and the patient of the action, a common strategy for reducing the ambiguity is to use SVO. Gibson and his colleagues conducted a series of experiments varying the reversibility and the complexity of events, by using embedded clauses, and asked their participants to pantomime events in three language groups: English (SVO), Japanese (SOV) and Korean (SOV) (Gibson et al., 2013). They found that with simple events, SVO speakers started to use SVO during gesturing the reversible events. However, SOV speakers remained consistent in their use of SOV, both for the reversible and the non-reversible events, and shifted to SVO only with more complex materials, when they had to gesture embedded clauses. In other words, the switch only happened when subjects had to describe grammatically more complex events, that is, when they had to start to grammaticalize in order to be able to easily describe them. Thus, Gibson et al.'s results, rather than with the reversibility of events, may be better explained by the hypothesis that during grammaticalization of the complex events, the preferred word order is SVO. This predicts the switch from SOV to SVO in the case of grammatically complex sentences, but not in the case of simple events (Langus and Nespor, 2010), independently from reversibility. Since in their experiment the events were complex, presumably participants started to use grammar in order to describe them. Furthermore, even though the authors do not report data regarding this question, there is a further possibility that during gesturing the grammatically more complex events participants started to use more consistent gestures, and less improvisation (similar to participants in the Hall et al., 2014 study), leaving enough cognitive resources for grammaticalization [which in turn yielded a preference for the syntactically unmarked SVO word order (Kayne, 1994)].

We propose that in the above mentioned cases, there might be an additonal factor that could explain a switch of word order preferences from SOV to SVO: participants started to use a consistent lexicon in the gesturing of the events. This consistency could emerge due to the explicit instructions of the task (in the Hall et al. study), or due to cognitive demands (Gibson et al., 2013). Thus, in order to gesture more complex events participants had to switch from improvised gesturing to a more stable and consistent gesture repertiore to be fluent during the task (in the Gibson et al. study)^1^. In other words, we hypothesize that the preference for either SOV or SVO emerged in these cases also depending on whether communication relied on improvised and spontaneously invented symbols, or on a stable and consistent lexicon.

We propose that the SOV and SVO orders reflect the preferences of two different cognitive systems: the conceptual system in direct interaction with the motor system, on the one hand, and the intervening computational system, on the other hand (Hauser et al., 2002; Pinker and Jackendoff, 2005; Langus and Nespor, 2010). While meaning (conceptual system), together with linguistic sounds or gestures (sensory-motor system) allows for simple communication, it is the function of syntax (computational system) to generate indefinitely long expressions from a finite set of elements by using the mechanism of recursion (Chomsky, 1957).

Since the conceptual system serves as a general and basic mode of communication, it prefers the SOV word order, which has been proposed by many authors as the order that emerged first in human languages (Givón, 1979; Newmeyer, 2000; Gell-Mann and Ruhlen, 2011). This view is supported by evidence reported about homesigners (Goldin-Meadow and Feldman, 1977; Goldin-Meadow and Mylander, 1998; Coppola and Newport, 2005), and about new sign languages, such as the Nicaraguan Sign Language (Senghas et al., 1997) or the Al-Sayyid Bedouin Sign Language (Sandler et al., 2005; Aronoff et al., 2008), where consistent SOV word order was found^2^. Furthermore, experiments on improvised gesturing also give evidence that when subjects have to invent gesture symbols, they use a consistent SOV order (e.g., Langus and Nespor, 2010).

In contrast, the computational system starts to operate only during grammaticalization. However, in line with previous evidence (Hudson and Eigsti, 2003), we propose that, due to memory and cognitive limitations, the computational system can operate only when lexical content items are easily accessible, i.e., when there is enough cognitive resource to express more complex grammatical structures. Thus, only in the presence of a lexicon, it is possible to use more elaborated syntactic structures, resulting in a preference for SVO. Hence, according to our prediction, a preference for the SVO order will arise when a stable and consistent lexicon is acquired.

Furthermore, we also suggest that once there are enough cognitive resources to grammaticalize, there is a default tendency to do so, since grammaticalization enables the expression of more complex structures in a cognitively more economical way, without the need of using case marking (that can clarify the role of grammatical categories of Subject, Object, and Verb even in the absence of a fixed word order)^3^. Consequently, we predict that while a preference for SOV arises in the case of spontaneous communication, by being exposed to a shared lexicon, which does not involve case morphology, there is a tendency to start using syntax. Furthermore, as a result, the general word order preference will switch to SVO (independently from either the semantic features, e.g., reversibility, or the complexity of the sentences).

We thus propose that under laboratory circumstances when people have to describe events by using gestures, if these gestures are part of a consistent gesture repertoire, there will be a tendency to use SVO, the preferred word order of the computational system of grammar. Furthermore, we propose that this switch takes place even if the gestured events are non-reversible, and they do not involve abstract concepts or complex structures (i.e., also in the case of simple sentences).

To test this hypothesis, we taught Italian (SVO) and Persian (SOV) speakers a set of gestures, and then asked them to describe simple events by using these gestures. According to our hypothesis, we expected to find a consistent use of SVO, independently from participants' native grammar.

## Methods

We chose our material (both the gestures and the events) from the Langus and Nespor study (2010), in order to be able to make a clear comparison between the gesture productions based on whether the gestures are improvised or previously acquired. In their study, the authors asked Italian and Turkish participants to describe simple events by using spontaneous gestures, and found that independently from their native grammar both groups used the SOV order during the description of the events (2010). Thus, in the present study, we used the same improvised gestures that Langus and Nespor recorded, and we also used the same pictures of events that they used in their study. However, whereas Langus and Nespor used in total 32 pictures of events, we decided to use only half of these events. The reason for this decision was that in our task subjects described the events with gestures that they first had to memorize, and we wanted to avoid that memory limitations would influence our results^4^. Our aim was to test whether due to the fact that participants were taught these gestures—and thus had a stable and consistent gesture repertoire that they could use fluently—they would switch from the SOV word order to SVO.

### Participants

Twenty-eight Italian (19 females, mean age 23) and 28 Persian adult native speakers participated in the study (10 females, mean age 26). The number of participants was chosen on the basis of the study of Langus and Nespor (2010) reported significant changes in word order preferences during their experiment. Italian participants were members of the School of Advanced Studies, Trieste, Italy. The Persian speakers were recruited from the Institute for Research in Fundamental Sciences (IPM), in Tehran, Iran. All subjects received a compensation of six Euros for their participation. They reported no vision or cognitive disabilities. None of the Italian participants were familiar with any SOV language, and similarly, none of the Persian speakers were familiar with any SVO language.

Our experiments were approved by the Bioethics Committee of the International School for Advanced Studies. This committee approved all the protocols of the present study. Participants were informed that the data obtained during the experiment would remain anonymous and there would be no report of individual data. Because of this and since we did not use any invasive or potentially harmful technique, we did not ask participants to sign a written consent form. Instead, after being informed about the instructions and the procedure of the study, we asked for participants' verbal consent to participate in the experiment. We only started the experimental procedure once we obtained this verbal consent. Thus, data recording of all participants also indicated their agreement.

### Materials

**Figure 1:**
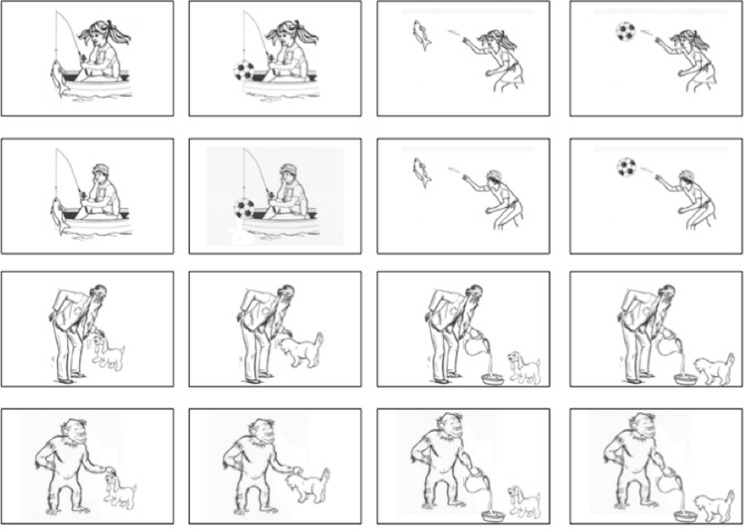
**Experimental material**. The sixteen vignettes depicting simple scenarios (from Langus and Nespor, 2010).

In the Teaching Phase, we used twelve movies where a woman gestured four agents (a man, a girl, an old man, and a monkey), four actions (stroking, feeding, throwing, and fishing) and four patients (a cat, a dog, a fish, and a ball), presented in random order. During the Test Phase we showed 16 vignettes, each of them depicting an agent performing an action with a patient. The 16 vignettes were either combinations of girl/man, fishing/throwing, ball/fish or combinations of old man/monkey, feeding/stroking, cat/dog (Figure 1).

### Teaching phase

During the Teaching Phase, participants were asked to learn a new language similar to sign languages, and were told that during the Test Phase they would have to describe each vignette by using the gestures they learned. In each teaching trial, participants saw two vignettes on the upper half of the computer screen along with a movie at the bottom of the screen. The two vignettes always had only one element in common. They could either depict (a) the *same agent* doing different actions with different patients, or (b) different agents doing the *same action*, with different patients, or (c) different agents, doing different actions, with the *same patient* (See S1 Movie for some samples of the movies of the Teaching Phase). The movie at the bottom of the screen, which accompanied the two vignettes, played repeatedly in a loop the gesture referring to the common part of the vignettes (i.e., it referred either to an agent, or to an act, or to a patient). Participants were instructed to memorize the gesture that referred to the common element of the vignettes. Thus, neither in the instruction of the teaching phase, nor during the presentation of the movies there was any reference to the categories of the gestures.

The movies were presented in a random order and each of them lasted as long as the subject pressed the space key in order to get to the next movie. The entire teaching session lasted as long as the participant thought s/he managed to learn all the gestures. Thus, participants could practice as long as they wanted. Before starting the Test Phase, we made a pre-test in order to see whether they could recall all the gestures without difficulties. In case someone made some errors or was uncertain about the gesture, we continued the Teaching Phase. Thus, the Test Phase started only when participants became confident and fluent in using the gestures. On average the Teaching Phase lasted approximately 10–15 min and we did not observe any differences regarding the length of the Teaching Phase between the two groups.

### Test phase

Participants saw a sequence of 16 vignettes on a computer screen presented in random order. These vignettes were the same they saw during the Teaching Phase. Participants were told that after the appearance of each vignette (that could remain on the screen as long as they needed), they had to describe it to the experimenter making the corresponding gestures. They were also explicitly told that each time they should use three gestures in order to describe all components of the vignettes, but these components (i.e., categories of agent, act, and object) were named in a random order for each participant, in order to avoid any influence on the order they would choose. Moreover, the experimenter also emphasized that during the description of the vignettes they could choose any order of the gestures. Our question was whether the chosen order was consistent throughout the experiment. Participants' answers were recorded both by a video camera and by the experimenter.

### Data analysis

We analyzed the proportion of the different orders of gestures participants used during test. Symbols referring to the acting persons were coded as Subject, symbols referring to acts as Verb, and symbols referring to the objects of the acts as Object. Thus, there could be a total of six different possible orders: SVO, SOV, OSV, OVS, VSO, and VOS. For each participant, we counted the number of times they used a specific order. We analyzed these proportions of word order variations in order to see the effect of task manipulation between the two linguistic groups and within each group.

## Results

### Results of experiment

**Figure 2:**
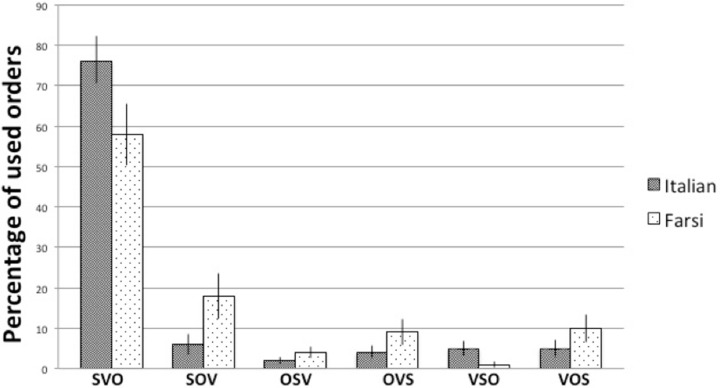
**Results of experiment**. Italian and Persian speakers' gesture strings for describing simple scenarios: distribution of constituent orders for Subject, Object, and Verb.

Since all of our participants managed to use the correct gestures during the task, we included all trials in the analysis. As predicted, both the Italian and the Persian group used SVO in the majority of the cases during gesturing. In the Italian group the distribution of the six orders was the following: SVO 76% (*SD* = 6.2), SOV 6% (*SD* = 2.6), OSV 2% (*SD* = 0.8), OVS 4% (*SD* = 1.8), VSO 5% (*SD* = 1.9), and VOS 5% (*SD* = 2.2). The Persian group gestured the following percentages of different word orders: SVO 58% (*SD* = 7.6), SOV 18% (*SD* = 5.6), OSV 4% (*SD* = 1.3), OVS 9% (*SD* = 3.1), VSO 1% (*SD* = 0.7), and VOS 10% (*SD* = 3.4) (Figure 2).

Since in both groups none of the other orders were more frequent than either SVO or SOV, and furthermore, these two orders were the crucial ones to our experimental question, we discarded the other types of word orders and calculated the proportions of the two critical orders in a way that they would sum up to a total of hundred percent. This resulted 88.5% SVO, and 11.5% SOV in the Italian group, and 72% SVO and 28% SOV in the Persian group. When we compared these proportions to a chance level, we found that in both groups the proportion of SVO was significantly above chance level [*t*_(27)_ = 10, 432, *p* = 0.0001 in the Italian group, and *t*_(27)_ = 3253, *p* = 0.003 in the Persian group]. Furthermore, we also compared between the two language groups the proportion of SVO answers they gave (SVO answers divided per total answers), and we found no significant difference [*t*_(27)_ = −1964, *p* = 0.06]. That is, even though Persian participants' native order is SOV, during the experiment they were not using significantly less SVO order than the native SVO speakers.

Thus, these results confirm our hypothesis that when participants have to use a previously acquired gesture repertoire, they prefer to use SVO, independently from their native language.

### Comparison between taught vs. improvised gestures

In order to see whether the acquisition of a gesture repertoire influenced participants' use of word order, we made a meta-analysis of our data and the data of Langus and Nespor (2010). Since both studies were designed in the same laboratory and except the instructions, used exactly the same method (including the same experimental material), this comparison allowed us to assess separately the effect of improvised vs. taught gestures. In the comparison, we only included gestural performance for those vignettes that were used bot in our experiment and in the study of Langus and Nespor (2010).

**Figure 3:**
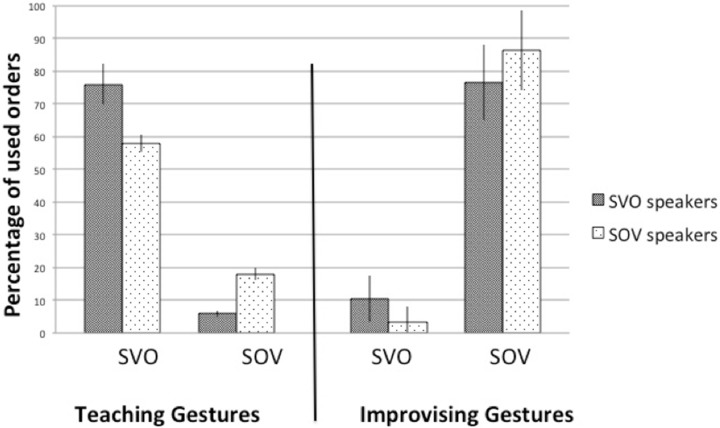
**Results of the meta-analysis**. Comparison between SVO and SOV speakers' gesture strings when the gestures were taught in our experiment vs. improvised in the experiment of Langus and Nespor (2010).

Figure 3 shows the proportion of SVO and SOV of the two language groups in our experiment and the experiment of Langus and Nespor [15].

To compare the two experiments, we carried out two separate Two-Way ANOVAs with two between-subject factors (Instruction and Language Group) and the dependent variables were the absolute proportions of SVO and SOV orders. Confirming our predictions, in both ANOVAs we found a main effect of Instruction [*F*_(1, 108)_ = 388.952, *p* = 0.0001, η^2^ = 0.783 in the case of SOV and *F*_(1, 108)_ = 144.086, *p* = 0.0001, η^2^ = 0.572 in the case of SVO] and another main effect of Language Group, [*F*_(1, 108)_ = 9.325, *p* = 0.003, η^2^ = 0.079 in the case of SOV, and *F*_(1, 108)_ = 6.840, *p* = 0.01, η^2^ = 0.060 in the case of SVO], presumably because native language still influenced word order choices. However, neither in the case of SVO answers, nor in the case of SOV answers we found a significant interaction between Instruction and Language Group [*F*_(1, 108)_ = 0.065, *p* = 0.800, η^2^ = 0.001 in the case of SOV and *F*_(1, 108)_ = 1.404, *p* = 0.239, η^2^ = 0.013 in the case of SVO], which means that independently from their native language, all participants reacted the same way to the experimental instructions.

To directly test our hypothesis that there would be a preference to use different word orders depending on whether participants have to use improvised or previously taught gestures, we made pairwise comparisons of the proportions of word orders between the two tasks. We found that SVO speakers used significantly more SVO word order during our experiment than during the experiment of Langus and Nespor [*t*_(54)_ = 10.327, *p* = 0.0001]. Furthermore, the proportion of SOV was also significantly higher during their improvised gesturing, compared to the present experiment, in which the gestures were taught [*t*_(54)_ = −20.592, *p* = 0.0001]. Similar results were found in the case of SOV speakers, who were also significantly more likely to use SVO when they had to first acquire the gestures [*t*_(54)_ = 7.029, *p* = 0.0001]. In contrast, when they were improvising their gestures, the proportion of SOV was significantly higher [*t*_(54)_ = −11.131, *p* = 0.020].

In sum, while in the experiment of Langus and Nespor (2010) both language groups used the SOV word order in the majority of cases, this pattern clearly changed and participants switched to SVO when they had to first acquire gestures and use them consistently in our experiment. These comparisons provide evidence that depending on whether participants use a previously acquired, stable gesture repertoire or they improvise their gestures, different word order preferences emerge regardless of the participants' native language.

## Discussion and conclusion

We hypothesized that depending on whether people spontaneously create communicative symbols, or use a previously acquired lexicon, preferences for different word orders emerge. Numerous studies have shown that improvised gesturing—i.e., communication without a pre-established lexicon—has a systematic order of gestures, which is always SOV (Gershkoff-Stowe and Goldin-Meadow, 2002; Goldin-Meadow et al., 2008; Langus and Nespor, 2010). In the present study, we showed that when a lexicon is given, SVO emerges: Persian participants changed their native language word order preferences and started to use SVO once they were taught a new lexicon.

The events in our study were non-reversible and did not trigger any syntactically complex structures^5^. Thus, our results can neither be due to any confusability between the agent and the patient (Meir et al., 2010; Gibson et al., 2013), nor to a role-conflict between the producer of the action-event and the agent of the action (Hall et al., 2013), The only factor that could have caused the switch in participants' word order preferences was the fact that they were using a previously learned consistent lexicon. While in the study of Langus and Nespor both Italian and Turkish participants used SOV during improvised gesturing, in our study, even though we used exactly the same material, when participants had to first acquire a complete gesture repertoire, they started to use the syntactically preferred SVO word order independently from their native grammar^6^.

A preference for SVO has been observed also in the case of creoles, languages created by children in pidgin communities. Pidgins originated in social situations where more mutually incomprehensible languages were spoken and speakers created a jargon not governed by grammar (Bickerton, 1984; Muysken, 1988). Children who had the lexical items of a pidgin as linguistic input created fully grammatical languages known as creoles. These languages are grammatically very similar across diverse geographical locations and are usually organized in the SVO order (McWhorter, 2001; Bakker, 2008). However, it has been difficult to determine why the SVO order emerges in Creole languages. For example, in Berbice Dutch, a systematic SVO order emerged from two SOV languages—Dutch and Ijo (Niger-Congo)—suggesting that the SVO preference is genetically determined (Kouwenberg, 1994). In contrast, other researchers have argued that Creole grammar, and especially the SVO order, can often be traced back to the source languages (DeGraff, 2003) and that there exist Creoles that allow additionally to SVO also other word orders (Escure and Schwegler, 2004).

We posit that throughout our experiment as well as those of Langus and Nespor (2010) we managed to create circumstances similar to those observed in the case of Creoles and homesigns, respectively. While Langus and Nespor asked their participants to describe simple events by using improvised gestures, our participants had to describe the same events with a previously learned gesture repertoire. Thus, we propose that the preference for the SOV order in the former case, and for the SVO order in the latter case are due to the same crucial difference observed between homesigns and creoles: the absence or the presence of a consistent lexicon.

We argue that our lexicon-based perspective reflects some inherent features of the human communicative systems, which can also be found in the case of these atypical language environments: those that give rise to Creoles and those that give rise to homesign. Due to general cognitive limitations, communication can either operate by using simple structures of spontaneous symbols (homesigns), or can generate indefinitely long expressions from a finite set of elements, once a stable symbol system is provided (Creoles).

While we believe that our proposal provides a strong explanation about the emergence of word order preferences, we acknowledge that the exposure to a shared lexicon may not be the only factor that influences word order preferences. For instance, Schouwstra et al. (2011), Schouwstra (2012), and Schouwstra and de Swart (2014) showed in their studies that during gesturing of extensional events (events including intensional verbs, in which the patient can be abstract or non-specific, or not existing at all), both Turkish and Dutch participants preferred to use the SVO order (Schouwstra and de Swart, 2014). The authors argue that while in extensional events (when actions are transitive, imply a motion in space, and the patients of the actions are usually concrete), patients and actions can be defined independently, and therefore the patient can occur before marking the action, intensional events should follow a different order, in which the actions have to precede the patient. Thus, these results give evidence that word order preferences can be also influenced by semantic factors, such as the abstractness of the described event.

In conclusion, while no explanation exists so far for the current diversity of word order preferences amongst natural languages, we believe that the results of our studies shed light on a very important factor that contributes to the emergence of word orders: the exposure to a shared lexicon. We consider this finding especially fruitful, because we think that by studying word order preferences in the laboratory, and by showing how different word orders emerge, we can also provide insights into the mechanisms and limitations of general cognitive processes underlying human communication.

### Conflict of interest statement

The authors declare that the research was conducted in the absence of any commercial or financial relationships that could be construed as a potential conflict of interest.

## Footnotes

^1^Similar results were found in the study of Langus and Nespor (2010) where both Italian and Turkish participants switched from SOV to SVO when instead of simple events they had to gesture embedded clauses.

^2^Note that if word order would be influenced by such factors as reversibility or complexity, we would expect these new sign languages to have SVO order as well.

^3^And as such, it usually characterizes SOV languages.

^4^In order to determine whether the results of the present study could be affected by the fact that we only used half of the vignettes used in Langus and Nespor (2010), we ran a meta-analysis on the original data by dividing Italian- and Turkish-speaking participants' preference for the SOV order into two groups: the preference for the SOV order for the 16 vignettes used in the present manuscript and the preference for the 16 vignettes disregarded in the present manuscript. A One-Way ANOVA showed that Italian-speaking adults' preference for the SOV order in the 16 vignettes used in the present study (SOV = 76.6%, SD = 11.5) and in the 16 vignettes we disregarded (SOV = 78.6%, SD = 7.6) did not reach significance [F_(1, 54)_ = 0.291, p = 0.45]. Also for Turkish-speaking participants' the difference between the 16 vignettes used (SOV = 86.4%, SD = 12.3) and the 16 vignettes disregarded (SOV = 92.2%, SD = 13.7) failed to reach significance [F_(1, 54)_ = 2.793, p = 0.10].

^5^Although the patients of the actions were animate in some of the cases (fish, dog, cat), neither during the teaching session, nor during the test phase were they introduced as potential agents. Thus, we exclude the possibility that any of them could have caused confusion or ambiguity during the task.

^6^Thus, contrary to the claim of Berwick et al. (2011), these results show that even in the case of gesturing, word order preferences depend on the presence of a lexicon.

## Supplementary material

S1 Movie **Examples of the movies we used during the teaching phase**.

S2 Dataset **The data collected during the experiment**.
